# Supplementary material for: Sea-Cucumber-like Microstructure Polyoxometalate/TiO2 Nanocomposite Electrode for High-Performance Electrochromic Energy Storage Devices
Source: Molecules. 2023 Mar 14;28(6):2634. doi: 10.3390/molecules28062634 (PMC10058481; doi:10.3390/molecules28062634)
Supplement: Supplementary file 1 [file molecules-28-02634-s001.zip › molecules-2265472-supplementary.pdf]

Supplement files

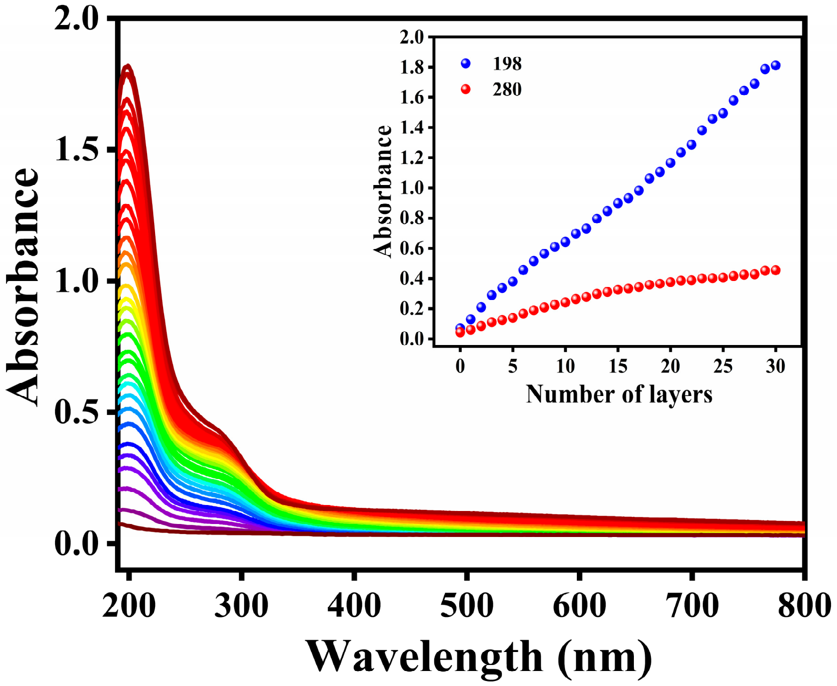

Figure S1. UV–vis absorption spectra of NW–$P_2W_{17}La$ film (number of cycles: 0–30). Inset: plots of the absorbance values at 198 and 280 nm.

UV–vis spectroscopy was used to monitor the layer-by-layer assembling process of the composite film owing to its facility in evaluating the growth process of the multilayers. As shown in Figure S1, the UV–vis absorption spectra of $P_2W_{17}La$ and PEI films (cycles: 0–30) assembled on quartz substrate. It can be seen that all the films exhibit the two characteristic absorption peaks at 198 and 280 nm in the UV region. The former is owing to the terminal oxygen to tungsten ($O_d \rightarrow W$) charge transfer transitions, and the latter is due to the charge transfer transitions from bridge-oxygen to tungsten ($O_b/O_c \rightarrow W$). In addition, the inset of Figure S1 shows the plots of the absorbance values at 198 and 280 nm as a function of the layer number, suggesting that the growth for each absorption cycle is uniform.

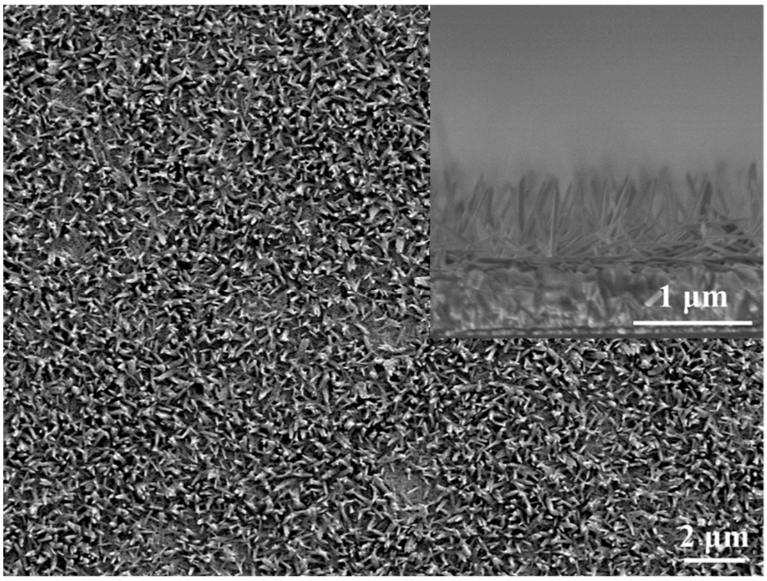

Figure S2. The SEM image of TiO_2 NW.

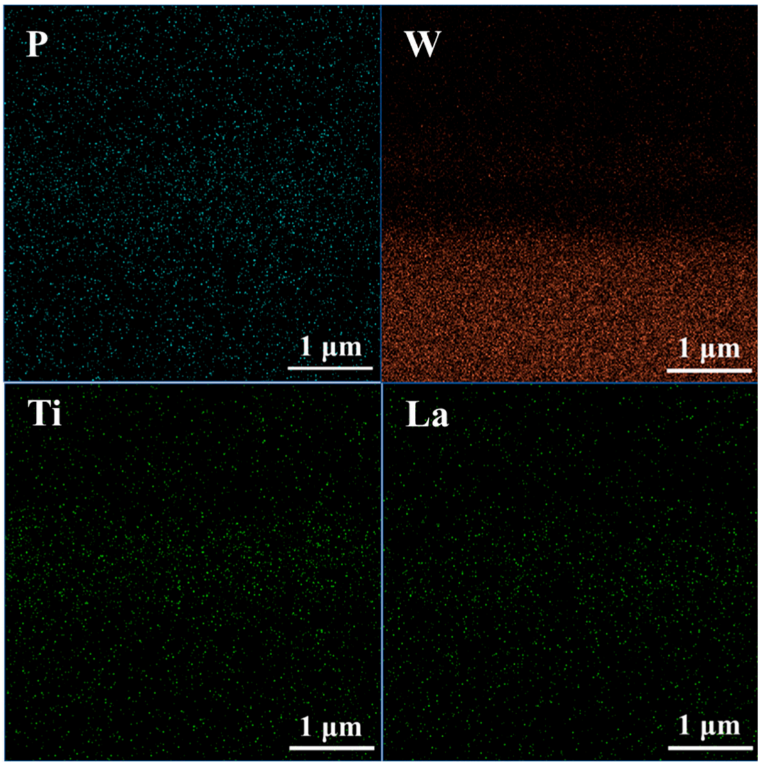

Figure S3. SEM-EDS mapping of NW–P_2W_{17}La for P, W, Ti and La. respectively.

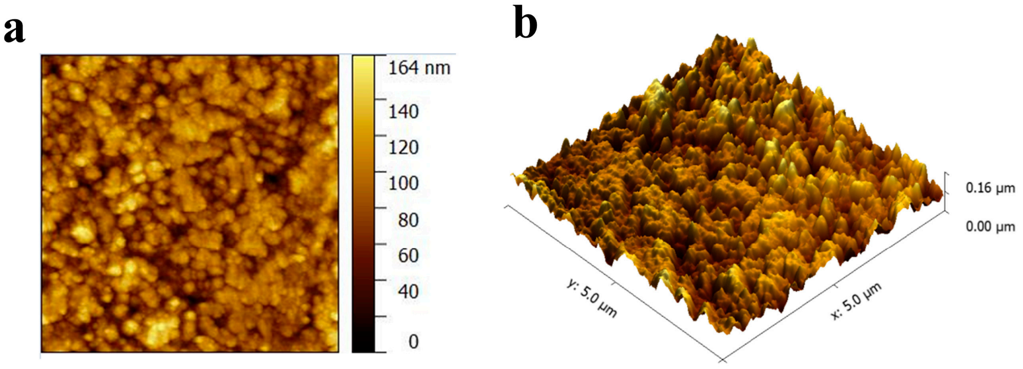

Figure S4. The 2D (a) and 3D (b) AFM images of FTO–P_2W_{17}La.

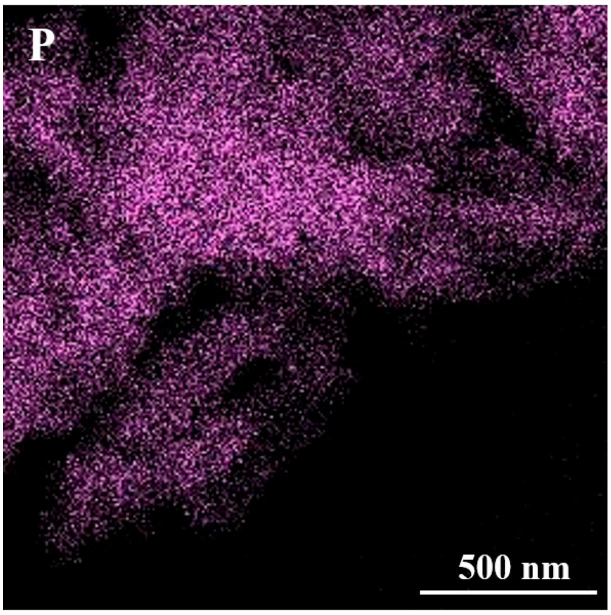

Figure S5. TEM-EDS elemental mapping patterns of P.

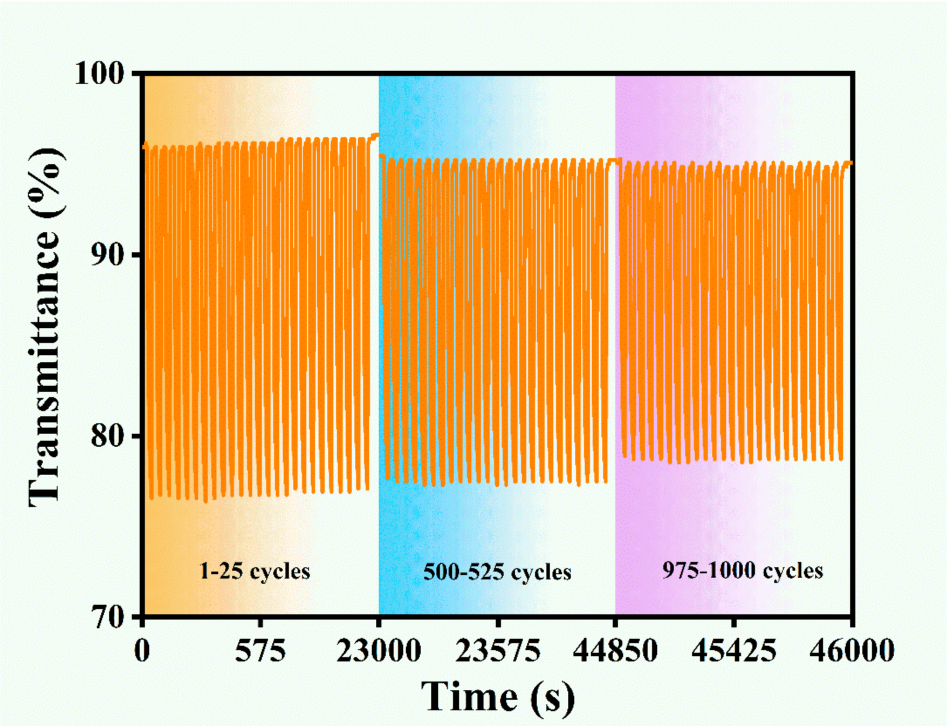

Figure S6. Cycle stability of FTO–$P_2W_{17}La$ film at 591 nm under square wave potentials of −1V and +1V.

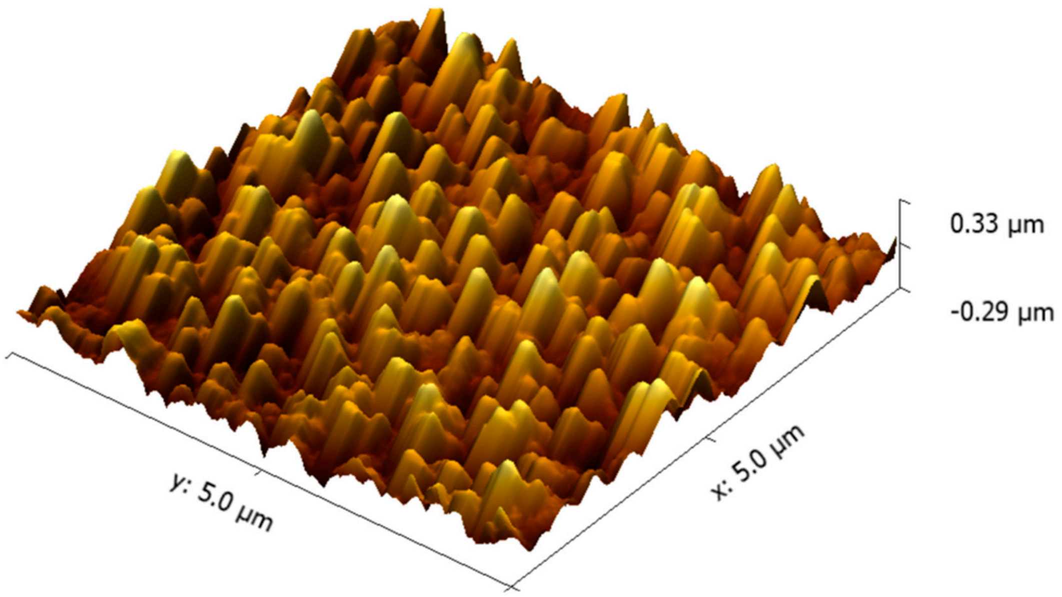

Figure S7. The AFM of NW–$P_2W_{17}La$ film after cycle stability test.

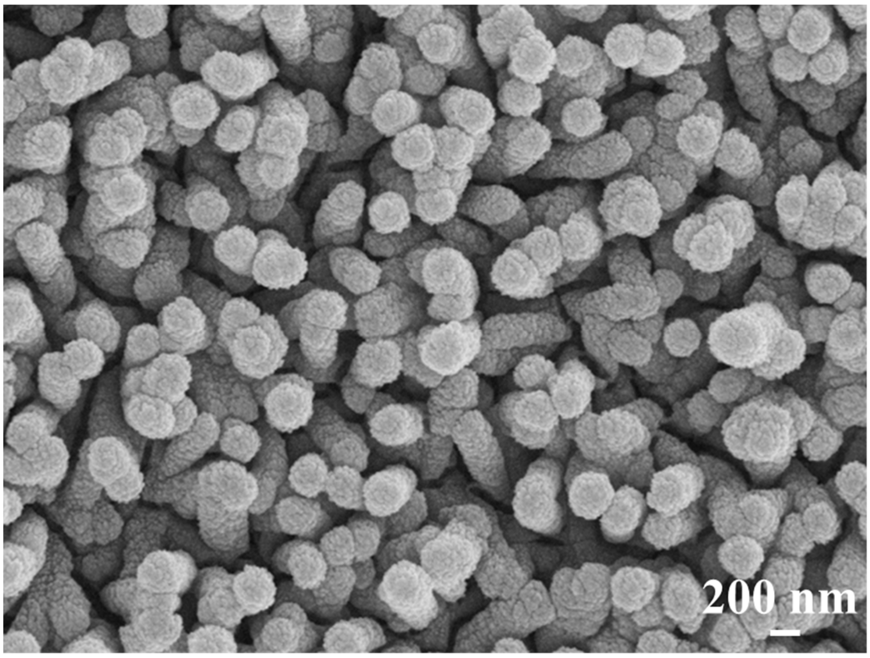

Figure S8. The SEM of NW–$P_2W_{17}La$ film after cycle stability test.

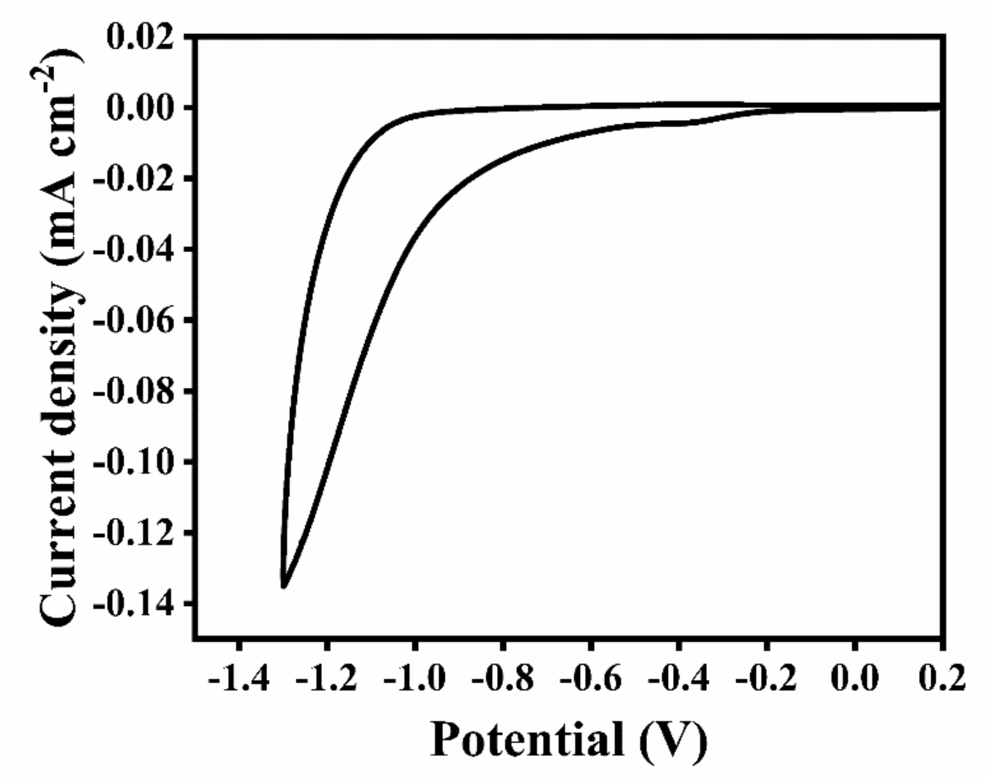

Figure S9. CV curve of TiO_2 NW film at a scan rate of 20 mV/s with $LiClO_4$/PC as the electrolyte.

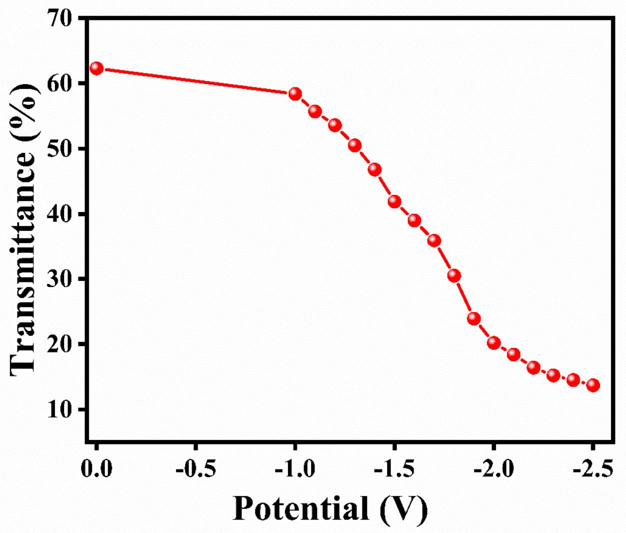

Figure S10. The transmittance changes of the EESD under different potentials.

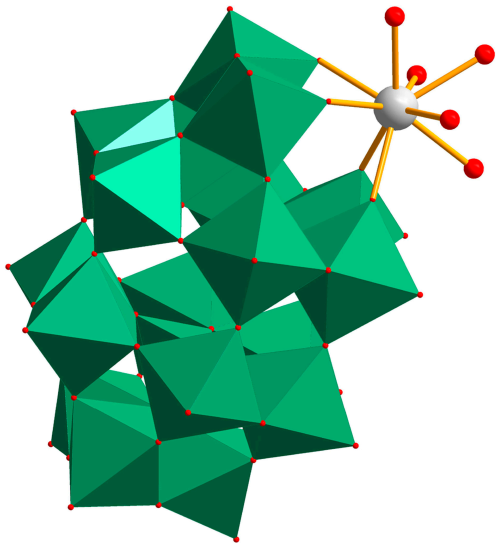

Figure S11. Polyhedral representation of $[La^{III}(H_2O)_x(\alpha_2\text{-}P_2W_{17}O_{61})]^{7-}$.

In our work, the lanthanide-containing monolacunary Dawson-type polyanions that we chose belongs to 1:1-type monomeric species, $[La(H_2O)_x(P_2W_{17}O_{61})]^{n-}$. The molecular formula of this type of POMs is $K_7[La(H_2O)_x(\alpha_2\text{-}P_2W_{17}O_{61})]$, but the crystal structure of which has not been confirmed by the X-ray diffraction method yet. According to the literature, in the structure of $[La^{III}(H_2O)_x(\alpha_2\text{-}P_2W_{17}O_{61})]^{7-}$, each La atom is nine-coordinate (mono-capped square antiprism) with four sites occupied by one $\alpha_2\text{-}P_2W_{17}O_{61}$ unit (as shown in Figure S10).

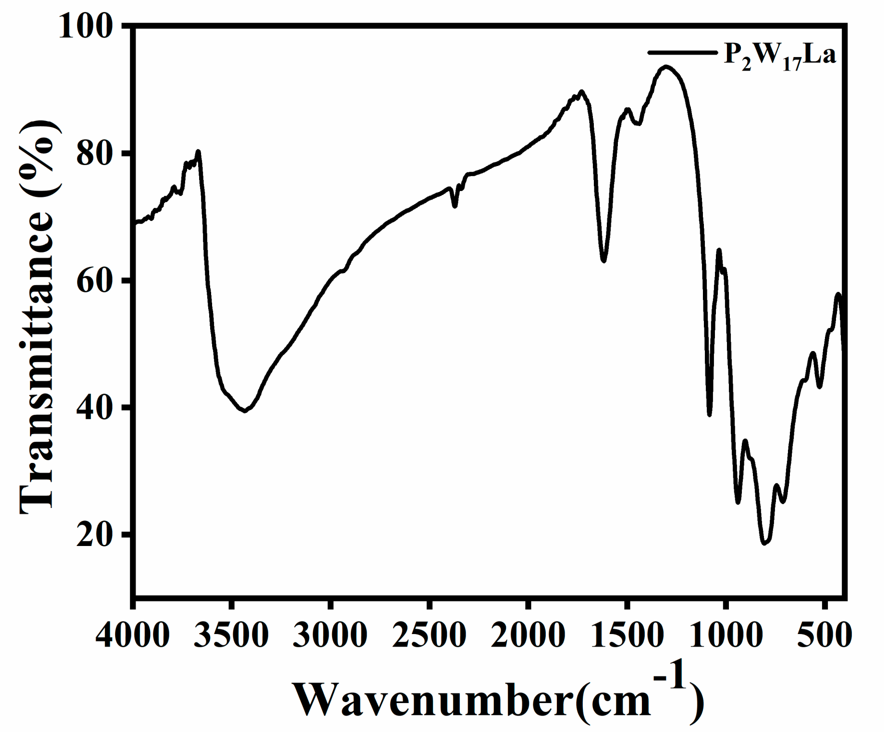

Figure S12. The IR spectra of $K_7[La(H_2O)_x(\alpha_2\text{-}P_2W_{17}O_{61})]$.

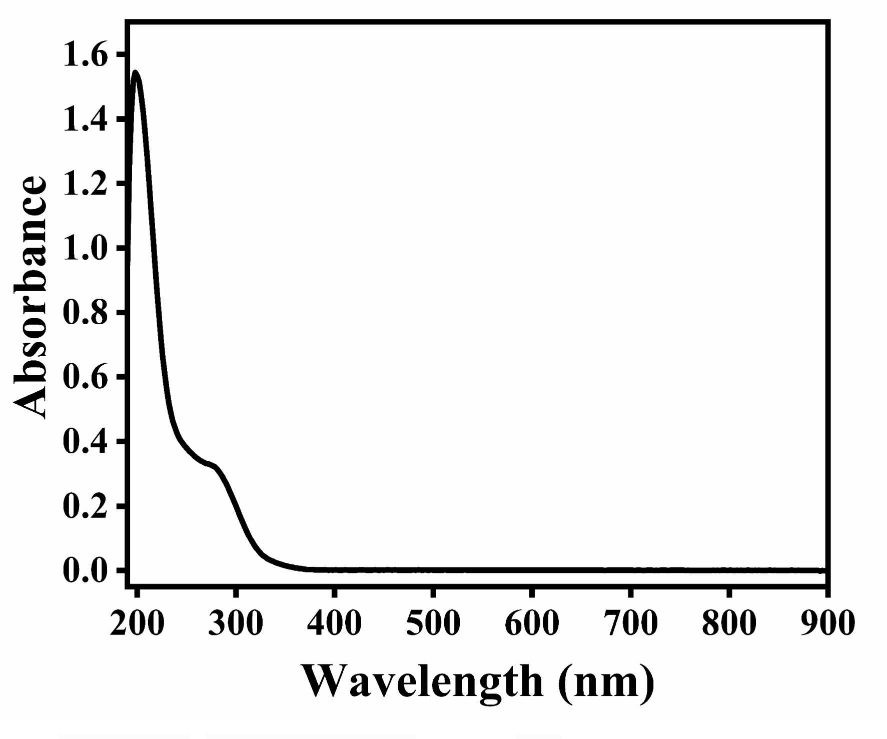

Figure S13. UV–vis absorption spectra of $K_7[La(H_2O)_x(\alpha_2\text{-}P_2W_{17}O_{61})]$.

The specific capacitance after material or EESD testing are calculated as follows:

$$C_V = \frac{I \times \Delta t}{\Delta \mathrm{V}} \qquad (1)$$

where C_V is the volume specific capacitance (mF·cm^{-3}), I is the discharge current density (mA·cm^{-3}), ΔV is the potential window (V), and Δt is the discharge time (s).

$$C_A = \frac{I \times \Delta t}{\Delta \mathrm{V}} \qquad (2)$$

where C_A is the areal specific capacitance (mF·cm^{-2}), I is the discharge current density (mA·cm^{-2}), ΔV is the potential window (V), and Δt is the discharge time (s).

Table S1 Comparison of electrochromic and energy storage performance in this work and previous works about POMs-based and inorganic metal oxides electrodes

Number	Materials	Optical modulation (%)	CE (cm^2 C^{-1})	t_c/t_b (s)	Specific capacitance	Ref.
1	NiO nanoflake	40	63.2	2.7/1.8	74.8 mF cm^{-2}	51
2	V_2O_5	67.01	28.57	34.4/20.9	–	21
3	$MnO_2/Ni(OH)_2$	53.8	34	2.66/2.72	28 mF cm^{-2}	16
4	Hybrid WO_3 nanoarrays	-	92.3	3.0/3.6	47.4 mF cm^{-2}	52
5	AgNW/WO_3	55.9	80.2	1.7/1.0	13.6 mF cm^{-2}	53
6	WO_3-V_2O_5	60	61.5	4.9/0.61	38.75 mF cm^{-2}	54
7	P_5W_{30}/PAH-Fe(phen)$_3$	35.17	94.73	2.49/0.9	10.45 mF cm^{-2}	32
8	NW/P_2W_{17}/Fe(phen)$_3$	34.3	194.5	2.8/6.2	135.8 F cm^{-3}	33
9	NW-P_2W_{17}	33.5	150.34	9.05/1.69	172.3 F cm^{-3}	55
10	NW-P_2W_{18}	45.1	69.0	1.9/6.7	–	56
11	NW-P_2W_{17}La	45.6	91.7	12/4	190.8 F cm^{-3}	Our work

NW: TiO_2 nanowire
